# A Review of Hematoma Components Clearance Mechanism After Subarachnoid Hemorrhage

**DOI:** 10.3389/fnins.2020.00685

**Published:** 2020-07-07

**Authors:** Pengjie Pan, Li Xu, Hongrong Zhang, Yuan Liu, Xiaocheng Lu, Gang Chen, Hailiang Tang, Jiang Wu

**Affiliations:** ^1^Department of Neurosurgery & Brain and Nerve Research Laboratory, The First Affiliated Hospital of Soochow University, Suzhou, China; ^2^Intensive Care Unit of Department of Anesthesiology, The First Affiliated Hospital of Soochow University, Suzhou, China; ^3^Department of Neurosurgery, Huashan Hospital, Fudan University, Shanghai, China

**Keywords:** subarachnoid hemorrhage, early brain injury, hematoma components clearance, Virchow-Robin space, hemoglobin

## Abstract

Subarachnoid hemorrhage (SAH) is a complicated clinical syndrome, which is caused by several kinds of cerebrovascular disorders, with high morbidity, disability and mortality rate. In recent years, several studies have shown that early brain injury (EBI) is an important factor leading to the poor prognosis of SAH. A major cause of EBI has been attributed that hematoma components invade into the brain parenchyma, resulting in neuronal cell death. Therefore, the clearance of hematoma components is essential in the clinical outcome of patients after SAH. Here, in the review, we provide a summary of the current known hematoma components clearance mechanisms and simultaneously propose a new hypothesis for hematoma components clearance.

## Introduction

Subarachnoid hemorrhage (SAH) is a serious cerebrovascular condition caused by bleed in the subarachnoid space. SAH may be caused by head injuries or ruptured cerebral aneurysms. Intracranial aneurysm rupture is the most important cause of SAH. SAH accounts for about 5% of all stroke types (Shah et al., 2019). Generally, the incidence rate of SAH is about 6–7/100,000 person/year (Linn et al., 1996; Anderson et al., 2000). However, Finland and Japan have higher incidence rates, about 20/100,000 person/year (Linn et al., 1996). The incidence rate of SAH in China has been reported to be 2/100,000 person/year (Anderson et al., 2000). The mortality rate of SAH ranges between 32 and 67% (Ingall et al., 2000; Nadeau et al., 2019).

Recent researches show that early brain injury (EBI) is a major factor for high mortality and disability after SAH. EBI refers to the period from initial bleeding to the onset of delayed cerebral vasospasm (within 72 h after SAH) (Broderick et al., 1994). The pathophysiological mechanisms for EBI include increased intracranial pressure (ICP), insufficient cerebral blood flow, oxidative stress, inflammation, neuronal apoptosis, necrosis, and autophagy (Chen et al., 2014; Rass and Helbok, 2019). When SAH occurs, ICP sharply elevates, and the increase rate is indicative of the severity of the initial bleed. Increased ICP will cause decreased cerebral blood flow and cerebral ischemia. After SAH, hematoma components invade brain parenchyma with cerebrospinal fluid (CSF), causing series of destructive reactions that lead to neuronal cell death. Hematoma components are mainly composed of red blood cells (RBCs), hemoglobin (Hb) and its lysate, etc. Hb and its lysis have strong cytotoxic effects that have been demonstrated to cause neuronal cell death (Zille et al., 2017). Hence, the removal of hematoma components plays a crucial role in the outcome of SAH patients. In clinics, the removal of hematoma is a treatment option including external ventricular drainage and lumbar cistern drainage. And these treatments have been proved to reduce mortality, improve survival, and enhance the life quality of patients after SAH.

## Neurotoxicity of Hematoma Components

**FIGURE 1:**
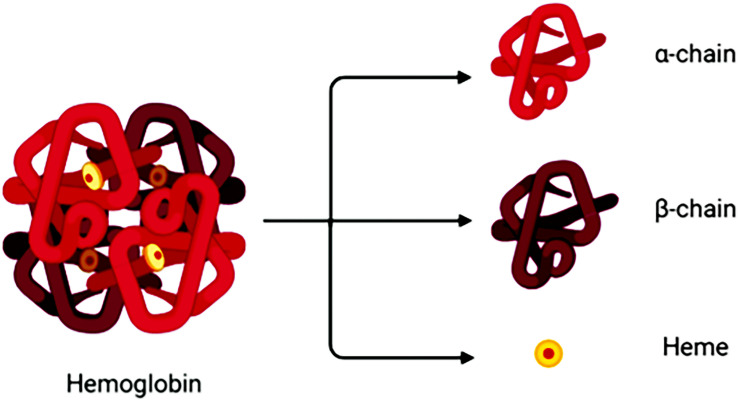
A hemoglobin molecule comprises two alpha (α1,α2) and two beta (β1,β2) subunits. Between each subunit fold is a hydrophobic pocket containing a heme group. Due to its four subunits, Hb molecules can reversibly bond with four O_2_ molecules.

**FIGURE 2:**
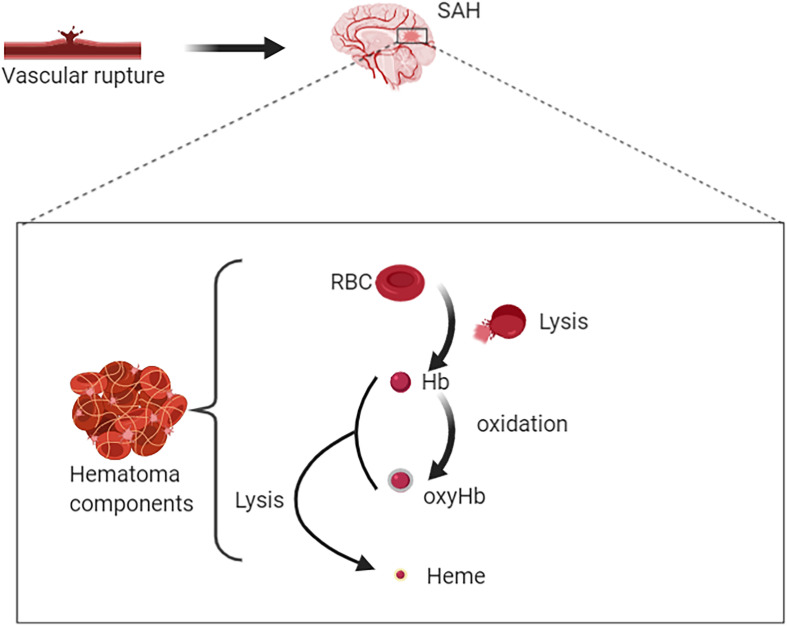
Rupture of intracranial blood vessels causes blood flow into the subarachnoid space to form subarachnoid hemorrhage (SAH). Then hematomas are formed after SAH. Hematoma components include intact red blood cells (RBCs), free hemoglobin (Hb), oxygenated Hb, free heme, and etc. RBC lysis produces free Hb. On the one hand, Hb can bind with oxygen molecules (up to four oxygen molecules) to form oxyhemoglobin; on the other hand, it could be lysed to produce free hemes.

Degradation of RBCs after SAH results in the release of Hb. Hb consists of four globin chains (α1, α2, β1, β2), with each containing a heme group (Figure 1). The heme group consists of a porphyrin ring that coordinates with an iron (Fe) atom in the Fe^2+^ or Fe^3+^ oxidative state. Released Hb can be oxidized to oxyHb. Released Hb cleavage produces free heme, which is considered to be more toxic than released Hb because of its lipophilic properties that enable its insertion into membranes (Balla et al., 1991). Moreover, free heme accelerates tissue damage through peroxidative reactions and activation of inflammatory cascades (Ma et al., 2016). Therefore, after SAH onset, the hematoma components are mainly composed of RBCs, oxyHb and free heme (Figure 2).

The toxicity of released Hb is manifested in four aspects: oxidation, inflammation, nitric oxide (NO) clearance, and edema (Bulters et al., 2018). Heme-mediated oxidation is in its ferrous (Fe^2+^) and iron (Fe^3+^) states, and heme can react with hydrogen peroxide or endogenous lipid hydroperoxide to form a highly reactive ferryl iron (Fe^4+^) (Bulters et al., 2018). The generation of these free radicals results in the dramatic modification of membranes, lipids, proteins, nucleic acids, and so on, which severely alters cell morphology and function (Marnett et al., 2003). Molecular markers of the oxidation process have been detected in the CSF after SAH, including covalently modified proteins and oxidized lipids (Suzuki et al., 1983; Reeder et al., 2002). Hb and heme are ligands of the Toll-like receptor 4 (TLR4), expressed by microglia and macrophages. Activation of TLR4 causes microglia and macrophages to secrete tumor necrosis factor (TNF), which triggers nuclear factor-κB activation, inflammation, and necrosis (Figueiredo et al., 2007; Kwon et al., 2015). Free heme can also activate the nucleotide-binding domain, leucine rich family and pyrin, leading to the synthesis of interleukin-1β (IL-1β) and interleukin-1α (IL-1α) by glial cells, leading to inflammation and neuronal cell death (Greenhalgh et al., 2012; Liu et al., 2019).

After SAH, ferrous Hb reacts with NO to produce methemoglobin and nitrate, thus losing its ability to bind and transport oxygen. On the other hand, NO is also consumed by oxygen free radicals (Joshi et al., 2002; Kajita et al., 1994). NO is a vasodilator produced by endothelial cells, neuronal cells, and glial cells in the brain. In cerebral blood vessels, NO regulates vascular tone and inhibits platelets adhesion (Faraci et al., 1994; Voetsch et al., 2004). In studies involving both human and animal models, it has been reported that NO depletion after SAH causes the dysfunction of coagulation system, which leads to thrombosis and is closely related to poor clinical outcomes (Suzuki et al., 1990; Stein et al., 2006; Friedrich et al., 2012). Hb and its lysate have been reported to induce brain edema as well. A study reported that intracerebral injection of Hb and its lysate resulted in increased sodium levels in the brains of rats (Huang et al., 2002). In another study, Hb injection into the rat brains resulted in the upregulation of matrix metalloproteinase-9 (MMP-9) and subsequent destruction of the blood-brain barrier (Katsu et al., 2010). Therefore, the occurrence of perivascular edema after SAH can be associated with Hb and its lysate.

As discussed above, the neurotoxicity of the hematoma after SAH gradually increases with the lysing of Hb. Therefore, the early clearance of hematoma has great significance. Hence, achieving hemoglobin clearance as early as possible and within a small range can greatly reduce neuronal cell death and have a positive impact on clinical outcomes.

## Progress of the Hematoma Components Removal Mechanism

**FIGURE 3:**
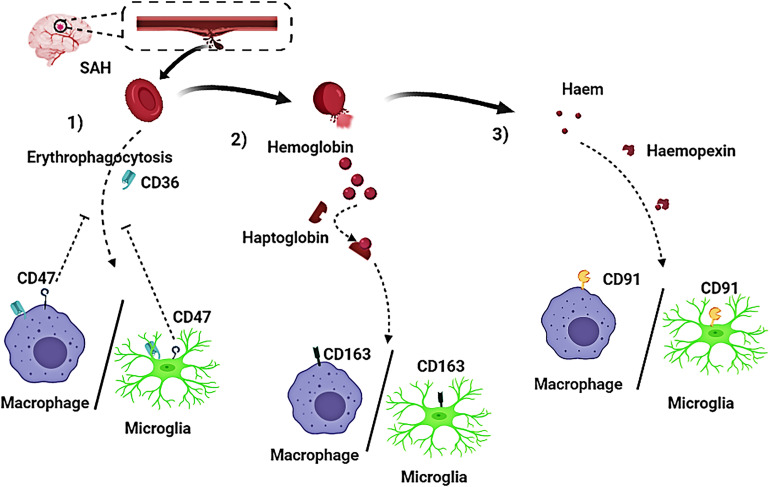
Activated macrophages and microglia play a crucial role in the clearing of hematoma components after subarachnoid hemorrhage (SAH). Three pathways have been reported on removal of hematoma components in patients with SAH. (1) Red blood cells (RBCs) are directly phagocytosed by macrophages/microglia after being recognized by CD36 receptors on macrophages/microglia membrane, and CD47 has an inhibitory effect on erythrophagocytosis; (2) The RBC cleavage product hemoglobin (Hb), firmly binds to haptoglobin (Hp) to form a haptoglobin-hemoglobin (Hp-Hb) complex by covalent binding. Subsequent recognition of the (Hp-Hb) complex by CD163 on the macrophage/microglia membrane mediates endocytosis of the complex; (3) Further lysing of Hb leads to the production of a large amount of heme or haem, which then binds to Hpx. This complex is transported into macrophages/microglia via CD91.

**FIGURE 4:**
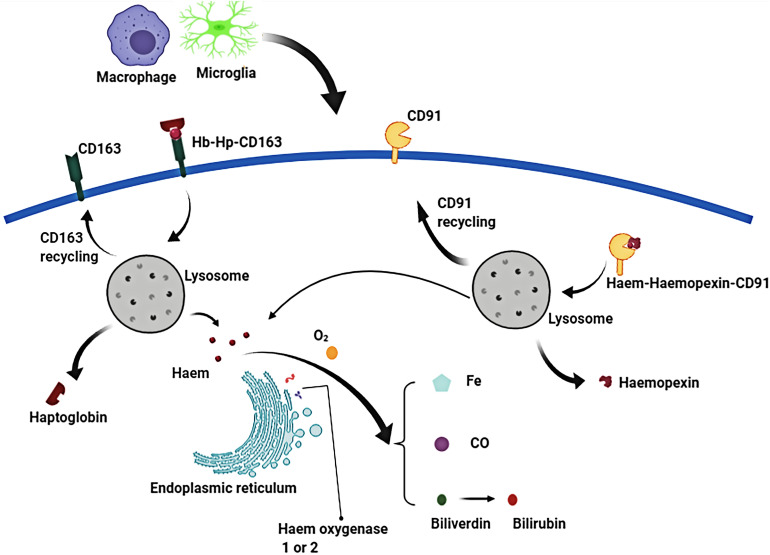
In the phagolysosome period, hemoglobin (Hb) is broken down to release heme or haem. Free heme is degraded to biliverdin, carbon monoxide (CO) and ferrous iron by the endoplasmic reticulum enzymes, haem oxygenase-1 or haem oxygenase-2. Biliverdin is reduced to bilirubin by biliverdin reductase. Similarly, free extracellular heme transported into macrophages/microglias is also broken down to produce free iron, biliverdin (bilirubin), and CO.

Currently, some research progress had been made regarding the mechanism of hematoma components clearance. Three approaches for the elimination of hematoma components have been reported and confirmed (Figures 3, 4):

### Erythrophagocytosis

Erythrophagocytosis is the clearance of abnormal RBCs. CD36 is an integral macrophage/microglia cell membrane protein and also a type II scavenger receptor expressed on macrophages and monocytes. Abnormal RBCs can exteriorize phosphatidylserine, and macrophages/microglia recognize exposed phosphatidylserine via cluster of differentiation CD36, leading to erythrophagocytosis (Fadok et al., 1998; Zhao et al., 2015). This shows CD36 play an key role in mediating recognition and phagocytosis. Erythrophagocytosis seems to be an effective clearance mechanism; however, once macrophages engulf more than two RBCs, they will undergo cell death, and lead to the release of deleterious heme and iron into the extracellular matrix. The role of CD47 in erythrocyte clearance is still under investigation. CD47 is an integrin-associated transmembrane protein that is expressed on lots of cell types including microglia, oligodendrocytes, and erythrocytes. It was reported that CD47 positively impacts on erythrocyte lifespan by inhibiting phagocytosis via signal regulatory protein alpha, which expressed on the surface of normal erythrocytes (Kondo et al., 1988; Ni et al., 2016; Tao et al., 2020). Further research is necessary to decipher the exact role of CD47 in erythrocyte clearance after SAH.

The fates of macrophages after phagocytosis of RBCs following SAH have not been thoroughly studied. Also, the possibility of hyperphagocytosis (involving more than two RBCs) by macrophages has not been studied in patients with SAH. Hence, erythrophagocytosis is not considered to be an ideal clearance mechanism.

### Haptoglobin and CD163

Erythrocyte cleavage produces Hb dimers that are immediately and irreversibly bound by haptoglobin (Hp), one of the strongest naturally occurring non-covalent interactions (Nagel and Gibson, 1971). A crystal structure analysis of the haptoglobin-hemoglobin (Hp-Hb) complex revealed active iron and pro-oxidative tyrosine residues on the Hp-Hb surface. Structural features of the Hp-Hb explain the ability of Hp to prevent Hb auto-oxidation and delay heme release (Andersen et al., 2012). Exposure of the epitope on the β-chain of Hp by the binding of Hb to Hp, allows the Hp-Hb complex to be recognized by CD163 on macrophages and initiates endocytosis. After internalization, heme is degraded by heme oxygenase 1 or 2 (HO-1 or HO-2) to biliverdin, iron, and carbon monoxide (Nielsen et al., 2007; Etzerodt et al., 2013; Schallner et al., 2015).

Haptoglobin is mainly synthesized by the liver and the reticuloendothelial system. Hp has been shown to diffuse from the blood into the CSF (Chamoun et al., 2001). It is worth noting that the amount of cell-free Hb is about 250 times greater than the amount of Hp in each milliliter of intracranial blood (Meng et al., 2020). Hp is the primary Hb-binding protein which can attenuate the adverse biochemical and physiological effects of extracellular Hb. Recent evidence indicated two major functions of the Hp-Hb complex: inhibition of Hb auto-oxidation (Buehler et al., 2009; Cooper et al., 2013; Arba et al., 2019) and Hb clearance (Kristiansen et al., 2001). There are two alleles and several known rare variants of Hp in humans (Kazmi et al., 2019). The two alleles are responsible for three kinds of possible genotypes with structural polymorphism: homozygous (1-1 or 2-2) and heterozygous (2-1) (Blackburn et al., 2018). Hp is cleaved into two subunits α and β, which are joined by a disulfide bond. Both alleles of Hp share the same β chain (Goldenstein et al., 2012). The β chain is responsible for the binding of Hb; thus, both genotypes have similar Hb binding affinity (Asleh et al., 2005). Clinical studies in patients with SAH indicated that Hp 2-2 patients may have high risk for hemorrhage-related complications and poor outcome (Blackburn et al., 2018).

Some studies had provided evidence that astrocytes (Lee et al., 2002) and oligodendrocytes (Zhao et al., 2009) express Hp under pathological conditions. CD163 is a phagocytic marker and Hb scavenger receptor, whose expression is thought to be exclusive to the perivascular and monocyte-macrophage system (Fabriek et al., 2005). Excessive levels of Hb upregulated the expression of Hp and the Hb-Hp receptor CD163 in neurons both *in vivo* and *in vitro* (Garton et al., 2016). CD163 mediates the delivery of Hb to macrophages/microglia after SAH. Macrophage/microglia endocytosis the Hp-Hb complex through CD163 when free Hb binds to Hp. Once the Hp-Hb complex is endocytosed by macrophages/microglia, the anti-inflammatory response may be fueled because of heme metabolites having potent anti-inflammatory effects (Moestrup and Moller, 2004).

CD163 is a single membrane-pass protein with nine extracellular domains, and also a member of the scavenger receptor cysteine-rich superfamily (Onofre et al., 2009). CD163 is involved in anti-inflammatory signaling following the binding of certain forms of Hp. This anti-inflammatory signaling includes the triggering of interleukin-10 (IL-10) responses via phosphatidylinositol-3 kinase-dependent Akt signaling (Galea et al., 2012; Landis et al., 2013; Wang et al., 2018). However, pro-inflammatory signals such as TNF-α, interferon-γ, transforming growth factor-β, and lipopolysaccharide could lead to decreased levels of CD163 expression.

Some researchers believed that CD163-mediated internalization of the Hp-Hb complexes into macrophages is vital in Hb clearance. However, some studies have shown that most of the Hb in the CSF after SAH does not bind to Hp (Galea et al., 2012). In addition, the Hp-CD163 pathway is not very effective in clearing Hb due to the scarcity of Hp. More importantly, it has been reported that neuronal cells also express CD163 under pathological conditions and mediate Hp-Hb endocytosis, leading to neuronal cell death (Chen-Roetling and Regan, 2016). In the absence of sufficient Hp reserve, the Hb structure is often modified by oxidation, thus reducing the ability of CD163 to bind to the Hp-Hb complex (Vallelian et al., 2008). It was reported that the level of Hp in the CSF of patients with SAH increased rapidly after blood was injected into the subarachnoid space. However, the Hp levels then decreased, possibly due to the clearance of the Hp-Hb complex. Subsequently, an increase in Hp levels accompanied by a parallel increase in Hb was observed, thus indicating a CD163-mediated clearance pathway saturation (Durnford et al., 2015; Przybycien-Szymanska et al., 2016). In summary, this pathway does clear Hb; however, reports from some studies have indicated that this pathway maybe is not the best one. Further research is needed to verify its role.

### Hemopexin and CD91

Hemopexin (Hpx) is a plasma glycoprotein, capable of binding heme with a high affinity and is expressed by neurons and glia (Morris et al., 1993; Paoli et al., 1999). A study reported that about 90% of Hpx in the brain is produced intrathecally under healthy conditions (Paoli et al., 1999). However, the level of Hpx in the CSF is ten times lower than that in the general circulation, indicating that its ability to bind to Hb in the brain is relatively low (Garland et al., 2016). The hemopexin-heme complex is endocytosed by cells expressing the low-density lipoprotein receptor-related protein-1 (LRP1)/CD91 receptor (Garland et al., 2016). LRP1 is a transmembrane receptor, which is expressed on macrophages, hepatocytes, neurons, vascular endothelial cells, pericytes, smooth muscle cells, and astrocytes (Paoli et al., 1999; Garland et al., 2016). The hemopexin-heme complex becomes internalized via endocytosis into cells upon binding to LRP1. The hemopexin-heme complex is then dissociated by lysosomal activity inside the cell. Heme is catabolized by heme oxygenase into biliverdin, carbon monoxide, and iron (Hvidberg et al., 2005). Study reported free Hb was still detected in the CSF after SAH (Garland et al., 2016), indicating that the hemopexin-CD91 system is not sufficient for SAH. In another study, one-third of the SAH patients had elevated levels of heme-binding proteins in the CSF, at an average of 133.8 μg/mL (Hvidberg et al., 2005). Patients with elevated levels of Hpx often have a higher incidence of delayed cerebral ischemia and worse functional outcomes compared with patients of normal heme-binding protein levels (Hvidberg et al., 2005). Thus, further investigation of this system is needed to despite its neuroprotective effects.

In addition to the above discovery, new research has shown that LRP1 can regulate the polarization of microglia through the Shc1/PI3K/Akt pathway during inflammation and oxidative damage (Peng et al., 2019). This causes microglia to become more responsive or pro-inflammatory, which is referred to as microglia priming. This means that LRP1 can promote the removal of hematoma components (including RBC and tissue debris), and inhibit the inflammatory response, thereby reducing damage to brain tissue after SAH.

## Our Hypothesis

We have proposed a possible Hb clearance pathway, which is effective and work in a small range. The details are introduced below.

### Virchow-Robin Space

**FIGURE 5:**
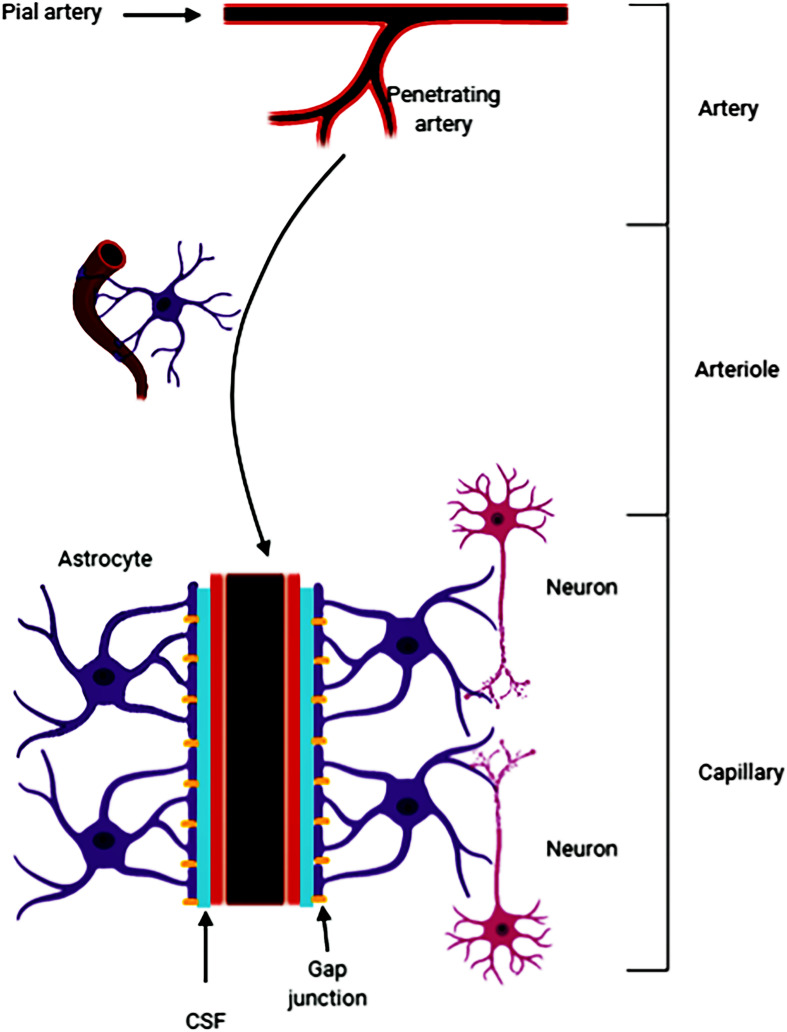
The pia mater becomes tapered and forms a capillary. The Virchow-Robin space (VRS) is the extravascular space around the small arteries and is full of cerebrospinal fluid (CSF). Astrocytes form gap junctions at the outer layer of the VRS, while the endothelial cells from the inner layer of the VRS. Astrocytes are sufficient to express aquaporin-4. The gap junctions facilitate the exchange of CSF and ISF. Substances in the CSF can enter the brain parenchyma through the VRS.

Fluid-filled canals surrounding perforating arteries, capillaries, and veins in the brain parenchyma are referred to the Virchow-Robin space (VRS) (Nakada et al., 2017; Plog and Nedergaard, 2018; Rasmussen et al., 2018). VRS was named after the first two scientists who described the structures in detail, Rudolph Virchow and Charles Philippe Robin. Then numerous studies were devoted to the deep understanding of the VRS. Research had shown that CSF (from the subarachnoid space) flows into the brain tissue through the perivascular spaces of the large leptomeningeal arteries (Iliff et al., 2013; Aspelund et al., 2015). Pial arteries in the subarachnoid space become smaller arteries that penetrate into the brain parenchyma (Ma et al., 2017; Pizzo et al., 2018). The space around the penetrating arteries which is filled with CSF is termed as the VRS (Zeppenfeld et al., 2017; Hannocks et al., 2018; Figure 5). In the VRS, CSF flows between blood vessels and glial cells, thus ensheathing the cerebral vasculature (Iliff et al., 2013). Some studies have suggested that astrocytes densely express aquaporin-4, which helps the CSF to flow into the brain parenchyma and mix with the interstitial fluid (ISF) for material exchange (Carare et al., 2008). A recent study reported that small volumes of soluble tracer injected into the ISF of the gray matter of the mouse striatum or hippocampus, led to the initial diffusion of the tracer through the extracellular spaces of the brain. However, within 5 min the tracer entered the basement membranes in the walls of the capillaries and cerebral arteries and drained out of the brain. The tracer was not retained within the brain parenchyma after injecting larger volumes of the tracer, suggesting that the tracer may have passed into the CSF in the ventricles (Morris et al., 2016). It is clear that substances within the CSF may potentially access and spread along the VRS to various extents throughout the cerebrovascular tree (Benveniste et al., 2017; Goulay et al., 2019). For example, large, full-length antibodies immunoglobulin G (IgG) have been shown to access the VRS of arterioles, capillaries, and venules following intrathecal infusion in rats (Kumar et al., 2018). It has been reported that solutes with molecular weights smaller than 100 kDa can leave the perivascular spaces by passing through the 50 nm clefts that separate the vascular endfeet of astrocytes (Jessen et al., 2015). Some studies have shown that perivascular macrophages and endothelial cells exist in the VRS (Iliff et al., 2012). In addition, the latest research found that CSF flows into the brain after stroke, causing acute tissue swelling (Mestre et al., 2020). Intracranial microvessel contraction and diffuse ischemia lead to an expansion of the VRS, and increases the flow rate of cerebrospinal fluid (Mestre et al., 2020).

In summary, since its discovery, an increasing amount of evidence have suggested that the VRS may play an important role in material transportation, waste removal (Iliff et al., 2012) and acute brain pathologies. However, the specific mechanism has not been elaborated yet.

### sCD163 and ADAM17

As discussed above, CD163 is an endocytic receptor for Hp-Hb complexes and is expressed on macrophages and monocytes. The extracellular portion of CD163 circulates in the blood as a soluble protein (sCD163) in healthy people. During inflammation and macrophage activation, sCD163 levels increase due to metalloproteinase-mediated cleavage (Etzerodt et al., 2010). However, the molecular mechanisms responsible for CD163 shedding are not fully understood. TNF α-converting enzyme (ADAM17/TACE) has been identified to cleave CD163 using metalloproteinase inhibitors and siRNA-mediated knockdown (Boretti et al., 2009). ADAM17, originally named TACE, is a membrane-anchored metalloproteinase, and expressed on macrophages and responds to thrombin and lysophosphatidic acid (Roy-O’Reilly et al., 2017). A study reported that sCD163 is elevated in the serum of patients with intracerebral hemorrhage (ICH) compared with healthy controls (Etzerodt et al., 2017). sCD163 synthesized intrathecally in patients with ICH, and accumulated in the subacute phase. The early serum levels of sCD163 in these patients were associated with hematoma and edema expansion following ICH (Etzerodt et al., 2017). Other studies have shown that sCD163 retains the ability to bind to the Hp-Hb complex and exert anti-inflammatory effects (Subramanian et al., 2013; Mohme et al., 2019).

### A Potential Pathway for Hemoglobin Clearance

**FIGURE 6:**
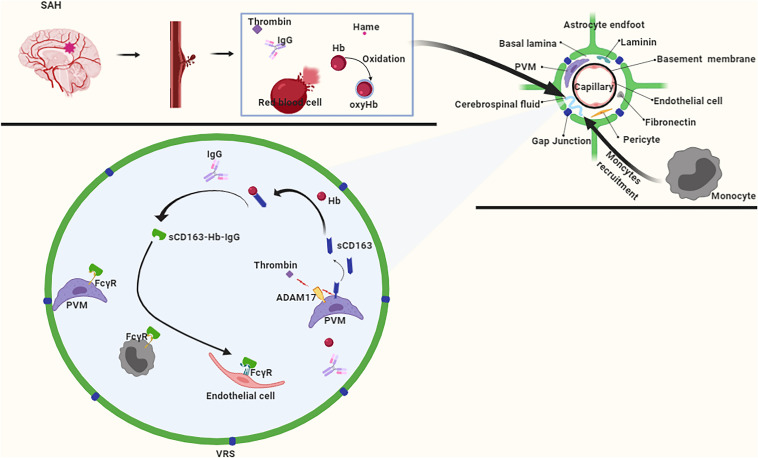
After subarachnoid hemorrhage (SAH), free Hb enters the VRS, and monocytes also enter VRS through recruitment. ADAM17 responds rapidly to thrombin stimulation, and CD163 is cleaved to produce a free form of CD163 (sCD163). After binding to hemoglobin (Hb), sCD163 inhibits the autoxidation of Hb on the one hand, and furtherly forms a complex with IgG on the other hand. The complex is recognized by macrophages, endothelial cells, and monocytes via the FcγR to achieve Hb clearance.

In the early stages of SAH, hematoma components enter the CSF and flow into the VRS. In the VRS, the hematoma component thrombin promotes ADAM17 activation, which further cleaves CD163 to produce sCD163 (Boretti et al., 2009), which forms a complex with Hb that inhibits the oxidation of Hb to oxyhemoglobin (Subramanian et al., 2013). Subsequently, the Hb-sCD163 complex binds to IgG to form the Hb-sCD163-IgG complex (Subramanian et al., 2013). On the one hand, the sCD163-Hb-IgG complex induces paracrine transactivation of neighboring endothelial cells, and causes them to upregulate HO-1 and secrete cytokines for systemic defense against Hb (Chen et al., 2010; Jovic et al., 2010). On the other hand, the sCD163-Hb-IgG complex elicits an autocrine loop of endocytosis via FcγR on perivascular macrophages (Chen et al., 2010; Jovic et al., 2010), whereas the internalized Hb is catabolized by HO-1. Also, due to the molecular weight of the Hb-sCD163-IgG complex being much larger than 100 KDa, it enters the brain parenchyma slowly through the VRS. This slow pace of the Hb-sCD163-IgG complex provides sufficient time for the recruitment of monocytes. Monocytes express FcγR (Subramanian et al., 2013), which enables them participate in the clearance of Hb as well (Figure 6).

However, our hypothesis has certain limitations. For example, the stability of the VRS is unknown after SAH. Moreover, brain edema, inflammatory reaction, and the blood-brain barrier are destroyed in the latter stages (Bosche et al., 2020). More importantly, astrocytes that form in the space around the VRS express aquaporin-4. Although its function is still controversial, it is likely to have a certain impact on the outcome of SAH. However, our hypothesis has great potential to remove Hb in the early stages following SAH. Moreover, it is likely to play a role in the clinical treatment of EBI following SAH.

## Conclusion

Removal of hematoma after SAH is important for the reduction of mortality and disability. The existing hematoma components clearance pathways have some limitations that cannot be overlooked. Therefore, it is essential to elaborate an effective hematoma components clearance pathway, which may provide new clinical treatment options for SAH. Thus, further research is required to provide more insights into the mechanism of hematoma clearance after SAH. Our perspective is that early, rapid, efficient, and low-toxic hematoma components clearance pathways should be clarified to benefit clinical treatment for SAH and improve the outcomes of patients.

## Author Contributions

PP drew the pictures. LX wrote the manuscript. HZ revised the manuscript. YL and XL collected the literatures. GC proposed the idea for the manuscript. HT and JW were responsible for instructing the manuscript. All authors contributed to the article and approved the submitted version.

## Conflict of Interest

The authors declare that the research was conducted in the absence of any commercial or financial relationships that could be construed as a potential conflict of interest.
